# *Gymnanthemum koekemoerae* (Compositae, Vernonieae), a new species from South Africa

**DOI:** 10.3897/phytokeys.36.7386

**Published:** 2014-04-30

**Authors:** Harold Robinson, Vicki A. Funk

**Affiliations:** 1 US National Herbarium, Department of Botany, NMNH, Smithsonian Institution, Washington, D.C. 20560 USA

**Keywords:** Asteraceae, Compositae, *Gymnanthemum*, South Africa, Vernonieae

## Abstract

A new species of *Gymnanthemum* (Compositae, Vernonieae) from South Africa is described. It can be distinguished from other species in the genus by the five-flowered capitula and widely obtuse leaf blades.

## Introduction

In the course of preparing a monograph covering all of the Vernonieae of southern Africa (Botswana, Namibia, South Africa) a specimen sent from PRE was determined to be a previously undescribed species of *Gymnanthemum*. Here we describe this new taxon, provide a key to the endemic species from South Africa, and an original illustration.

## Taxonomic treatment

### Gymnanthemum

Cass.

http://species-id.net/wiki/Gymnanthemum

Gymnanthemum Bull. Soc. Philom. Paris 1817: 10. 1817. Type: Gymnanthemum cupulare Cass. = *Gymnanthemum coloratum* (Willd.) H. Rob. & B. Kahn
Bracheilema R. Br. ex Salt, Abyss. Append.: 65. 1814, nom. nud.
Decaneurum DC., Arch. Bot. (Paris) 2: 516. 1833, nom. superfl., type same as *Gymnanthemum*.
Plectreca Raf., Fl. Tellur. 4: 119. 1838. Type: *Staehelina corymbosa* Thunb.
Keringa Raf., Sylva Tellur.: 144. 1838. Type: *Vernonia amygdalina* Del.
Cheliusia Sch. Bip. in Hochstetter, Flora 24(1, Intelligenzbl.): 26. 1841, nom. nud.
Vernonia subsect. *Urceolata* S.B. Jones, Rhodora 83: 67. 1981. Type: *Vernonia sphaerocalyx* O. Hoffm.

#### Remarks.

Shrubs or small trees moderately to densely branching; stems often felted, hairs rarely asymmetrically T-shaped. Leaves alternate; petioles short, winged or elongate; blades membranaceous to rather coriaceous, margins entire to serrate or dentate, upper surfaces essentially glabrous and somewhat glossy to arachnoid tomentose. Inflorescences terminal, densely corymbiform, with small bracteoles; peduncles short; involucral bracts coriaceous, 25–35, 4–5-seriate, inner bracts often deciduous. Florets 3–50; corolla white to violet, anther base broadly tailed, tails often long, apical appendage glabrous; style base without or with scarcely distinct node; style branches with stout, pointed sweeping hairs. Achenes 5–10-costate, raphids short, elongate or not evident; pappus of many rather persistent capillary bristles, often with broadened tips, with outer series of short squamellae. Pollen sublophate. Chromosome number *n* = 10, 20. More than 43 species are found in sub-Saharan Africa, Madagascar, Southern Asia, and also introduced into Brazil.

The genus *Gymnanthemum* was described by Cassini (1817), included in *Vernonia* by Candolle (1836) and Bentham (1873) and resurrected by Robinson and Kahn (1986) and Robinson (1999). The generic limits have changed and are now more narrow than in 1999. Currently the genus has nine species in southern Africa (Robinson et al. in prep.), five of which are endemic to South Africa; a key to those is provided here. The four more widespread species are *Gymnanthemum theophrastifolium* (Schweinf. ex Oliv. & Hiern) H. Rob., *Gymnanthemum coloratum* (Willd.) H. Rob. & B. Kahn, *Gymnanthemum amygdalinum* (Del.) Sch. Bip. ex Walp. and *Gymnanthemum myrianthum* (Hook. f.) H. Rob. The still unfinished monograph will cover all species of Vernonieae from Southern Africa with descriptions, keys and pollen images (Robinson et al. in prep.).

### Gymnanthemum koekemoerae

H. Rob. & V.A. Funk sp. nov.

urn:lsid:ipni.org:names:77138105-1

http://species-id.net/wiki/Gymnanthemum_koekemoerae

#### Type.

**Figure 1.**
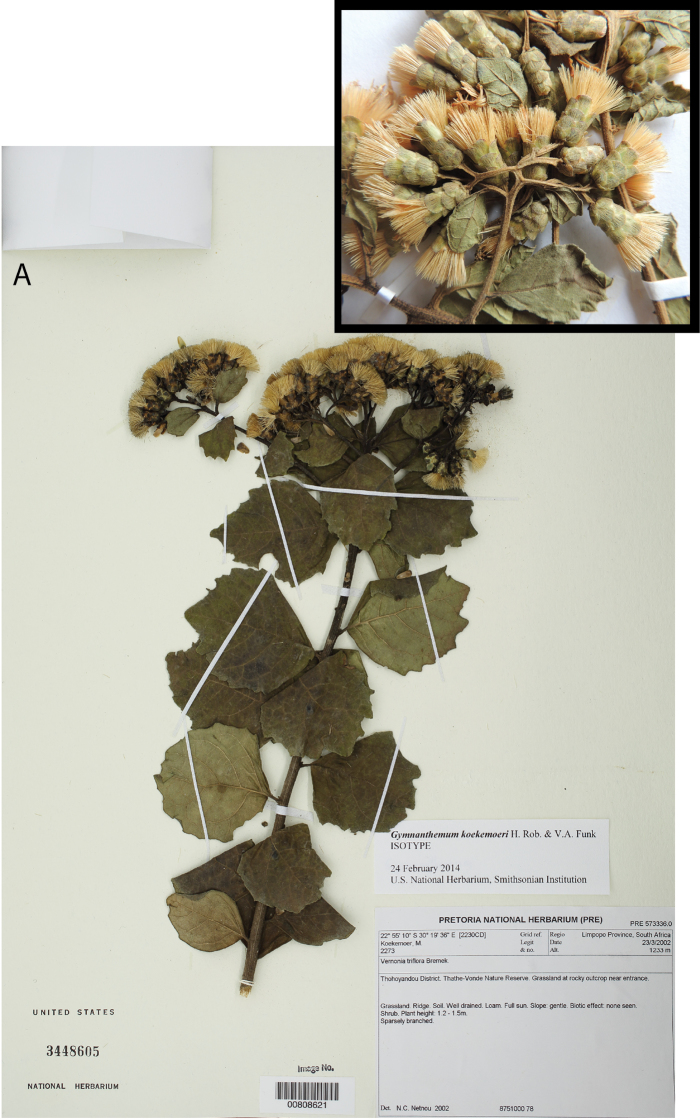
Type specimens. **A** Photograph of the isotype (US) **B** Photograph of the inflorescence of the holotype (PRE).

**Figure 2.**
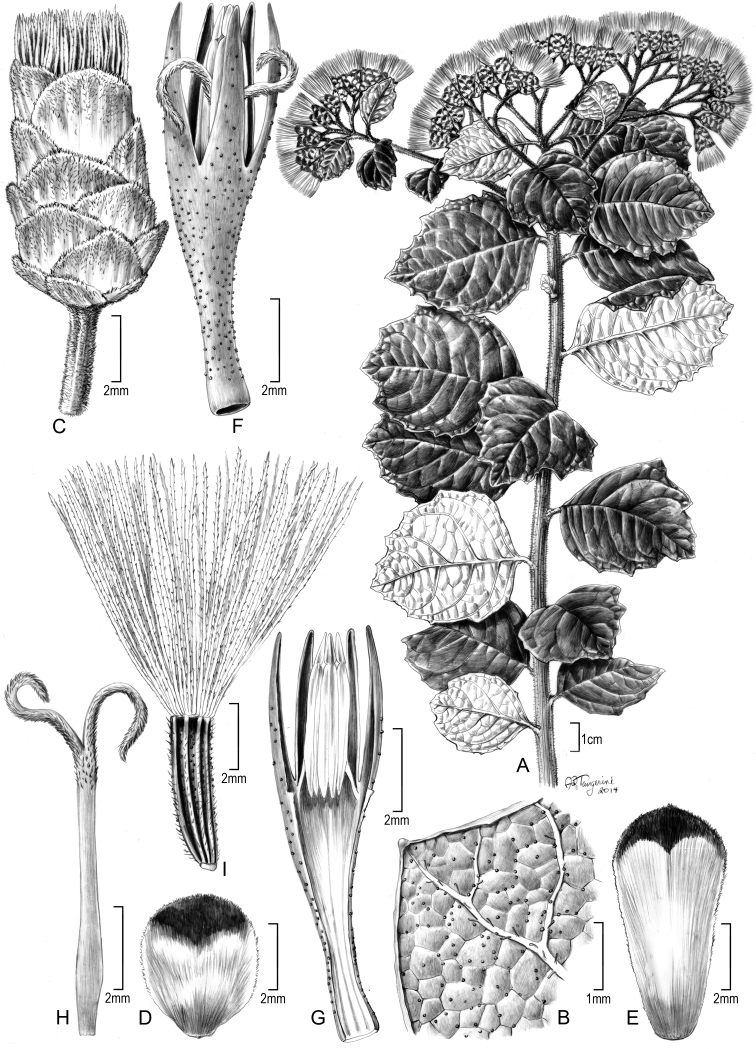
Original Illustration of *Gymnanthemum koekemoerae* H. Rob. & V.A. Funk: **A** Habit **B** Abaxial surface of leaf **C** Head **D** Outer involucral bract **E** Inner involucral bract **F** Floret **G** Longitudinal section of floret showing anthers **H** Style **I** Achene with pappus. [Illustration by Alice Tangerini (US)]

**Figure 3.**
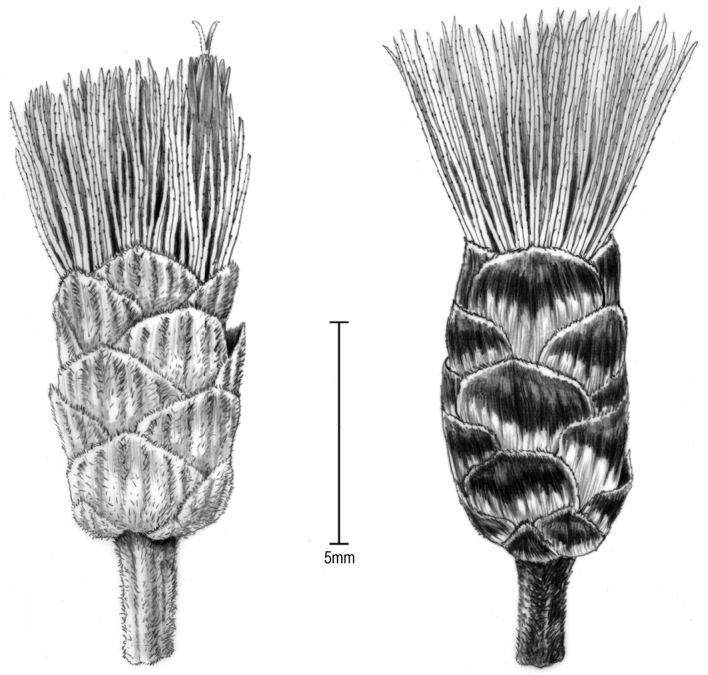
Illustration of heads from the holotype: **A** Head when in flower **B** Head with mature achenes. Note: there is an increase in dark coloration on the more mature head, possibly caused by fungi. [Illustration by Alice Tangerini (US)]

South Africa. Limpopo Province. Thohoyandou District. Thathe-Vonde Nature Reserve. Grassland at rocky outcrop near entrance, 1233 m, 22°55'10"S, 30°19'36"E [2230CD], 23 March 2002, *Koekemoer 2273* (holotype PRE!, isotype US!). Figs 1–3.

#### Description.

Sparsely branched shrubs 1.3–1.5 m tall; stems brown, terete and striate, hispid to hirtellous and gland-dotted, hairs unicellular, with short branches and spurs. Leaves alternate; petioles 3–4 mm long; leaf blades chartaceous, suborbicular, 4.5–6.5 cm long and broad, bases rounded to broadly obtuse, abruptly terminating at petiole, margins with c. 5 broad dentations above basal ¼, apex with broad obtusely triangular tip; adaxial surface dark green when dry, essentially glabrous, primary and secondary veins priminulous in shallow grooves, tertiary veins flush with surface; abaxial leaf surface somewhat paler, sparsely pilosulous on larger prominulous veins, surface with numerous yellow glandular dots; secondary veins widely spreading at 50–80° angles, usually 4 on each side, quaternary veins minimally prominulous. Inflorescence broadly corymbiform, terminal on stems and distal branches; peduncles 3–8 mm long, capitula 13–15 mm high; involucres 4–5 mm wide, to 7–8 mm wide when in fruit; involucral bracts subimbricate in c. 5 gradate series, round to oblong, 2–7 mm long, 2.5–3.6 mm wide, inner bracts somewhat ranked, apices broadly rounded to subtruncate, with broad rounded surface outside, greenish or brownish with darker and gland-dotted distal 1/4; florets 5 in a capitulum; corollas pale lavender, c. 9.5 mm long, essentially without hairs, sparsely gland-dotted outside, basal tube c. 5 mm long, funnelform distally, throat c. 0.5 mm long, lobes evenly tapered, c. 4 mm long; anther thecae c. 4.5 mm long, apical appendage triangular, c. 0.6 mm long, 0.25 mm wide; achenes c. 5 mm long, 10-ribbed, with numerous short, spreading setulae mostly on ribs, with glandular dots between ribs; pappus mostly c. 9 mm long, becoming tawny, of c. 90 crowded capillary bristles, bristles scarcely broadened distally.

#### Related taxa.

*Gymnanthemum koekemoerae* is closest to *Gymnanthemum mespilifolium* in its leaf pubescence, but it has an abrupt base on the leaf blade, totally unlike the narrow acumination in *Gymnanthemum mespilifolium* that gives the leaves of the latter a long-petiolate appearance. The blades of the new species are also more chartaceous, and the dentations of the leaf are more numerous and are as broad as long. The dentations in *Gymnanthemum mespilifolium* are long and narrowly acute, and are restricted to the distal 1/3 of the leaf blade.

#### Notes.

The holotype (PRE) has both flowering and fruiting material while the isotype (US) material is mostly fruiting. The specimen of the new species was distributed as *Vernonia triflora* Bremek. (now *Gymnanthemum triflorum* (Bremek.) H. Rob.) which has only 3 florets in its capitula, has stiffly and densely hispid stems, and has ovate to oblong leaf blades with hispidulous abaxial surfaces.

#### Etymology.

The species is named for Dr. Marinda Koekemoer (PRE) who collected the type material and who has done so much to further our knowledge of the Compositae of southern Africa.

#### Distribution.

This species is known only from the type locality.

### Key to the endemic species of *Gymnanthemum* from South Africa

**Table:** 

1	Abaxial surface of leaves sparsely puberulous to essentially glabrous	2
–	Abaxial surface of leaves hispid to tomentose	3
2	Leaf blades chartaceous with broadly obtuse bases; stems puberulous often with dark hairs (fungus), especially in fruiting specimens	*Gymnanthemum koekemoerae* H. Rob. & V.A. Funk
–	Leaf blades rather membranaceous with long-acuminate bases; stems essentially glabrous	*Gymnanthemum mespilifolium* (Less.) H. Rob.
3	Leaf blades oblong to ovate with obtuse bases; stems hirsute; capitula with 3 florets	*Gymnanthemum triflorum* (Bremek.) H. Rob.
–	Leaf blades obovate to oblanceolate with cuneate bases; stems tomentose; capitula usually with 4–5 florets	4
4	Stems and abaxial surfaces of leaves completely covered with appressed tomentum; inflorescence narrowly corymbose	*Gymnanthemum corymbosum* (L. f.) H. Rob.
–	Stems with tomentum of cottony hairs, abaxial surfaces of leaves with mixed erect and arachnoid hairs that do not totally obscure green surface; inflorescence broadly corymbose, much broader than high	*Gymnanthemum crataegifolium* (Hutch.) H. Rob.

## Supplementary Material

XML Treatment for Gymnanthemum

XML Treatment for Gymnanthemum koekemoerae
